# 2024 The Royal College of Physicians of Thailand (RCPT) clinical practice guidelines on management of dyslipidemia for atherosclerotic cardiovascular disease prevention

**DOI:** 10.2478/abm-2024-0033

**Published:** 2024-12-16

**Authors:** Praween Lolekha, Weerapan Khovidhunkit, Chaicharn Deerochanawong, Nuntakorn Thongtang, Thananya Boonyasirinant, Chatchalit Rattarasarn, Aurauma Chutinet, Vuddhidej Ophascharoensuk, Nicha Somlaw, Surapun Sitthisook, Surajit Suntorntham, Wannee Nitiyanant, Rungroj Krittayaphong

**Affiliations:** Department of Medicine, Thammasat University, Patrhumthani 12121, Thailand; Department of Medicine, King Chulalongkorn Memorial Hospital and Chulalongkorn University, Bangkok 10330, Thailand; Department of Medicine, Rajavithi Hospital and Rangsit University, Bangkok 10400, Thailand; Department of Medicine, Siriraj Hospital, Mahidol University, Bangkok 10700, Thailand; Department of Medicine, Ramathibodi Hospital, Mahidol University, Bangkok 10400, Thailand; Department of Medicine, Chiang Mai University, Chiang Mai 50200, Thailand; The Royal College of Physicians of Thailand, Bangkok 10320, Thailand

**Keywords:** atherosclerosis, cardiovascular disease, dyslipidemia, guideline, Thai

## Abstract

**Background:**

The Royal College of Physicians of Thailand (RCPT) published a Clinical Practice Guideline on Pharmacologic Therapy of Dyslipidemia for Atherosclerotic Cardiovascular Disease (ASCVD) Prevention in 2016. The availability of newer classes of medications for dyslipidemia, supported by extensive clinical research findings, indicates a significant need for the updating of the existing clinical practice guideline.

**Objectives:**

To serve as guidelines on the management of dyslipidemia for Thai adults.

**Methods:**

The RCPT Dyslipidemia Guidelines Committee was established with representatives from selected professional societies to revise the 2016 Guideline by critically reviewing the latest evidence. Meetings were conducted from August to December 2023, culminating in a public hearing that engaged various stakeholders in January 2024. The final Thai version received approval in April 2024, while the English translation was completed in October 2024.

**Results:**

Lifestyle modifications and statins remain the cornerstone of therapy for dyslipidemia in adults across various clinical settings. Emerging evidence regarding newer classes of lipid-lowering medications indicates that these treatments are effective in lowering LDL-cholesterol levels and reducing atherosclerotic cardiovascular events. This suggests that they may serve as an add-on therapy for individuals who cannot achieve target levels or who are at high risk for future cardiovascular events. The Thai CV Risk Score is recommended due to its specificity for the Thai population.

**Conclusions:**

The 2024 updated clinical practice guidelines establish a framework, provide recommendations, and serve as a comprehensive resource for the contemporary management of dyslipidemia in adults, with the goal of preventing ASCVD in Thailand.

Dyslipidemia is a common problem in the clinical practice of internists and general practitioners [1]. It serves as a significant causal risk factor for atherosclerosis, particularly in conjunction with other cardiovascular risk factors such as type 2 diabetes, hypertension, and cigarette smoking [2, 3]. Various forms of dyslipidemia exist, with the most common types including hypercholesterolemia, hypertriglyceridemia, low high-density lipoprotein-cholesterol (HDL-C) levels, and mixed dyslipidemia [2]. High low-density lipoprotein-cholesterol (LDL-C) level is now firmly established as the main cause of atherosclerotic cardiovascular disease (ASCVD) [3]. The atherosclerotic process begins at an early age with the accumulation of cholesterol in the vascular endothelium [4], a phenomenon facilitated by the presence of other cardiovascular risk factors and genetic predisposition. The process occurs over decades without any symptoms until the atherosclerotic plaque grows large enough to encroach upon the coronary artery lumen, causing luminal stenosis that leads to angina pectoris [4]. The acute complication is caused by the rupture of atherosclerotic plaque, which exposes the subendothelial content to the bloodstream and triggers clot formation, leading to subtotal or total coronary artery occlusion typical of acute coronary syndrome (ACS) [5]. This concept can also be applied to ischemic cerebrovascular disease and peripheral arterial disease.

Numerous clinical studies have demonstrated that lowering LDL-C levels is associated with a reduction in the risk of future cardiovascular events in patients with chronic coronary syndrome, ACS, and cerebrovascular disease [6]. Consequently, clinical practice guidelines from various professional societies have endorsed LDL-C lowering as a primary treatment strategy to mitigate cardiovascular risk in both primary and secondary prevention settings [2, 5]. Examples of these clinical practice guidelines are the 2019 European Society of Cardiology (ESC) guidelines for the management of dyslipidemia [2], the 2018 American Heart Association (AHA)/American College of Cardiology (ACC) guidelines on the management of blood cholesterol [7], and the 2021 ESC guidelines on cardiovascular disease prevention [8]. Despite ongoing debate regarding the choice between a treat-to-target approach and the administration of high-intensity statins to all patients at intermediate or high cardiovascular risk, recommendations for the optimal LDL-C levels necessary to reduce cardiovascular risk have shifted toward more intensive LDL-C lowering therapies [7, 8]. Several meta-analyses have indicated that achieving lower LDL-C levels correlates with improved patient outcomes [9].

In 2016, the Royal College of Physicians of Thailand (RCPT) synthesized clinical evidence regarding the management of dyslipidemia into a set of recommendations and subsequently published a Clinical Practice Guideline on Pharmacologic Therapy of Dyslipidemia for ASCVD Prevention for clinical practice in Thailand. The guideline also considered several factors, including the context of local practice, the integration of recommendations from existing clinical practice guidelines, the availability of medications, and the various reimbursement schemes in Thailand. In recent years, the emergence of novel classes of medications for the treatment of dyslipidemia, supported by robust clinical evidence, underscores the necessity for an updated revision of the existing clinical guideline. The objective of the updated guidelines is to provide a framework for the modern management of dyslipidemia in adults, aiming to prevent ASCVD in Thailand.

## Guidelines development

The 2024 RCPT Clinical Practice Guidelines on Management of Dyslipidemia for ASCVD Prevention was developed by a committee appointed by the RCPT, consisting of medical specialists from various fields related to atherosclerosis and dyslipidemia. The clinical evidence was searched using PubMed, OVID, Web of Science, and Google Scholar, covering the past 10 years. The recommendations are derived from a comprehensive review of scientific evidence, expert opinions, and public consultations with relevant physicians and stakeholders. The strength of recommendations in the context of clinical practice in Thailand, along with the grading of these recommendations and the quality of evidence, is presented in **Tables 1–3**. The committee tasked with developing the guidelines operated independently in formulating the recommendations. Furthermore, any conflicts of interest among the committee members involved in the guidelines development were documented and disclosed. The guidelines were developed, appraised, and reported in accordance with the Appraisal of Guidelines for Research and Evaluation II (AGREE II) recommendations.

**Table 1.** The strength of recommendations in the context of clinical practice in Thailand

**Symbol**	**Definition**
++	“Strongly recommend” indicates a high level of confidence in the recommendation, as the measure is highly beneficial to patients and cost-effective.
+	“Recommend” indicates a moderate level of confidence in the recommendation, as the measure may be beneficial to patients and may be cost-effective in specific situations (it may be optional depending on the circumstances and appropriateness).
+/−	“Neither recommend nor against” indicates an uncertain level of confidence in providing the recommendation, as there is insufficient evidence to support or oppose the measure. It may or may not be beneficial to patients and may not be cost-effective, but it does not increase harm to patients. Therefore, the decision to proceed depends on other factors (it may or may not be done).
−	“Not recommend” indicates a moderate level of confidence in advising against the measure, as it is not beneficial to patients and is not cost-effective unless necessary.
−−	“Strongly not recommend/against” indicates a high level of confidence in advising against the measure, as it may cause harm or pose a danger to patients.

**Table 2.** Classes of recommendations

**Class**	**Definition**
I	The evidence and/or consensus suggest that the treatment or measure is beneficial, useful, and effective for patients.
IIa	The evidence or opinion tends to support the idea that the measure is beneficial and potentially effective for patients.
IIb	The evidence or opinion regarding the benefit and potential effectiveness of the measure is still unclear.
III	The evidence or overall consensus suggests that the treatment or measure is not useful, not effective, and in some cases, may be harmful to patients.

**Table 3.** Levels of evidence

**Level**	**Definition**
A	Data derived from multiple randomized clinical trials or meta-analyses.
B	Data derived from a single randomized clinical trial or large non-randomized studies.
C	Consensus of the experts and/or small studies, retrospective studies, and registries

### Definition

**Dyslipidemia** refers to abnormalities in lipoprotein metabolism, resulting in changes in the levels of various types of lipids in the blood, which become a risk factor for atherosclerosis.

**Cardiovascular disease** can be divided into:

- **Clinical ASCVD:** Refers to patients who currently have or have had ACS, myocardial infarction, stable coronary artery disease, stable/unstable angina, coronary or other arterial revascularization, ischemic stroke, transient ischemic attack (TIA), peripheral artery disease, or atherosclerotic aortic diseases.
- **Subclinical ASCVD:** Refers to individuals who do not yet show symptoms of ASCVD but have evidence of atherosclerosis detected through examinations, such as the presence of atherosclerotic plaque in coronary arteries, carotid arteries, or other large arteries, or a high coronary calcium score, among other findings.

**Atherosclerosis** refers to a condition caused by the thickening of the inner arterial walls due to the gradual accumulation of fatty cholesterol deposits, and sometimes calcium, leading to arterial narrowing.

**Primary prevention** refers to the treatment of individuals with abnormal blood lipid levels to prevent clinical ASCVD in those who do not yet have the disease.

**Secondary prevention** refers to the treatment of individuals who already have clinical ASCVD with the goal of preventing recurrent events.

## Behaviors and risk factors associated with the development of atherosclerosis

Atherosclerosis is the primary underlying cause of cardiovascular disease. This complex process often begins in childhood and progresses with age. The etiology of atherosclerosis involves a combination of genetic and acquired factors, which can be categorized into modifiable and non-modifiable risk factors, as summarized in **Table 4**.

**Table 4.** Behavior and risk factors associated with cardiovascular disease from atherosclerosis

**Lifestyles**	**Biochemical characteristics**	**Personal characteristics**
- Smoking - Poor nutrition - Physical inactivity	- Hypertension - Dyslipidemia - Prediabetes/diabetes - Obesity/visceral obesity - Sleep apnea - Hypercoagulable state/hemoconcentration - Inflammation marker - Proteinuria - Left ventricular hypertrophy	- Older age - Male sex - Family history of premature atherosclerosis - Poor socioeconomic status

**Modifiable factors**	**Non-modifiable factors**
- Smoking - High plasma glucose - Obesity - Physical inactivity - Hypertension - Dyslipidemia - Poor nutrition - Sleep apnea - Hemoconcentration - Proteinuria - Left ventricular hypertrophy	- Age - Sex - Genetics

### Principles of dyslipidemia screening

1. It is recommended to measure lipid levels in the following populations:
  - Individuals aged 35 years and above
  - Individuals with clinical ASCVD or subclinical ASCVD
  - Individuals at risk for cardiovascular diseases, such as those with diabetes or chronic kidney disease (CKD)
  - Individuals suspected of having familial hypercholesterolemia (FH) from physical examination, such as the presence of xanthoma at the eyelids, elbows, knees, palms, thickening of the Achilles tendon, or arcus cornea in individuals younger than 45 years.
2. It is recommended to measure total cholesterol (TC), triglycerides (TG), and HDL-C during the initial screening for dyslipidemia. LDL-C is calculated using the Friedewald formula, expressed in milligrams per deciliter (mg/dL):
LDL-C(mg/dL)=TC(mg/dL)−HDL-C(mg/dL)−[TG(mg/dL)/5]
3. It is recommended to use direct LDL-C measurements in cases where TG levels are greater than or equal to 400 mg/dL or when LDL-C levels are <50 mg/dL.
4. LDL-C should be the primary value used to assess cardiovascular risk and to establish treatment targets, whether calculated or measured directly.
5. It is recommended to calculate non-HDL-C in individuals with high TG levels, diabetes, or obesity.
Non-HDL-C(mg/dL)=TC(mg/dL)−HDL-C(mg/dL)

### Guidelines for plasma lipid testing

Plasma lipid tests that do not require fasting include:

1. Initial assessment of cardiovascular risk.
2. Monitoring treatment outcomes.
3. Testing non-HDL-C levels.

Plasma lipid tests that require fasting (except for 50 mL of water) for at least 10–12 h include the following:

1. Before starting treatment for dyslipidemia in the case of primary prevention.
2. For individuals suspected of having genetic dyslipidemia or those with a family history of premature ASCVD.
3. When non-fasting TG levels are >400 mg/dL.

### Cardiovascular disease risk assessment

Assessing the 10-year cardiovascular risk is crucial for effective treatment planning, including setting goals and choosing appropriate lipid-lowering medications. Various evaluation criteria have been developed from studies conducted in diverse populations across different countries and are referenced in numerous guidelines, such as the Framingham Risk Score, Pooled Cohort Equation, Systematic Coronary Risk Evaluation (SCORE), and QRESEARCH cardiovascular risk algorithm (QRISK). These tools differ slightly in the factors they use to calculate cardiovascular risk.

Clear evidence shows that the likelihood of future cardiovascular events varies across ethnic groups. In Thailand, it is recommended to use risk scores specific to the Thai population, such as the Thai cardiovascular (CV) Risk Score. This score can be calculated with or without blood test results; in cases where blood tests are unavailable, height and waist circumference can serve as substitutes. The Thai CV Risk Score is derived from data collected in a long-term cohort study on cardiovascular and metabolic disease risk factors among employees of the Electricity Generating Authority of Thailand (EGAT Heart Study) [10].

The 10-year Thai CV Risk Score is available via a mobile application. A score <10% indicates low risk, while a score of 10% or higher suggests moderate to high risk. Other risk scores derived from Thai population studies may also be applicable if validated through accurate risk prediction studies.

### Principles of dyslipidemia management

Disorders in lipoprotein metabolism can lead to alterations in plasma lipid levels, which may result in ASCVD, ultimately causing disability, hospitalization, or death. Consequently, the principles of dyslipidemia treatment are focused on preventing atherosclerosis. Both primary and secondary preventions can be achieved by addressing various risk factors associated with atherosclerosis, including controlling blood pressure, managing plasma glucose levels in diabetic patients, quitting smoking, and treating dyslipidemia.

The management of dyslipidemia can be achieved through lifestyle modifications and the appropriate use of lipid-lowering medications. The principles guiding the treatment of dyslipidemia are as follows:

1. Initial blood tests and laboratory investigations: Conduct blood tests and initial laboratory investigations prior to initiating treatment to assess the specific type of dyslipidemia.
2. Screening for genetic dyslipidemia: Investigate suspected cases of genetic dyslipidemia, such as FH, in patients with a personal or family history of premature ASCVD (defined as occurring before age 55 years in men and 60 years in women) or those exhibiting xanthomas upon examination.
3. Identifying secondary causes: Look for factors that may promote or contribute to dyslipidemia, such as nephrotic syndrome, hypothyroidism, or Cushing syndrome. Dyslipidemia in these patients may normalize with the treatment of the underlying conditions.
4. Assessing ASCVD: Evaluate whether the patient has prior ASCVD. If present, the objective of dyslipidemia is aimed at secondary prevention. In some cases, history and physical examination alone may not be sufficient, and additional laboratory tests may be necessary, such as those for asymptomatic myocardial infarction or abdominal aortic aneurysm in obese individuals. If there is evidence of previous ASCVD, the patient is considered very high risk, and potent lipid-lowering medications should be used even if plasma lipid levels are not highly elevated. The treating physician must be aware of the efficacy of each lipid-lowering medication and its suitability for the patient's risk level (Appendix 1).
5. Risk assessment for patients without prior ASCVD: In patients aged 35 years and older without a history of ASCVD, assess the 10-year cardiovascular risk using the Thai CV Risk Score. If medication is indicated, select the appropriate type and dosage to optimize benefits, safety, and cost-effectiveness.
6. Lifestyle modification counseling: Advise patients on lifestyle modifications, such as dietary changes, appropriate exercise, and smoking cessation. These measures not only improve plasma lipid levels but also reduce other associated risk factors, such as lowering blood pressure and controlling plasma glucose in diabetic patients. This may lead to reduced medication dosages, lower costs, and avoidance of adverse drug reactions.
7. Setting plasma LDL-C targets: Establish individualized LDL-C targets based on the patient's cardiovascular risk for both primary and secondary prevention. This approach guides appropriate treatment to achieve the desired target levels. However, attaining the LDL-C target is influenced by various factors beyond medication, including genetics, lifestyle modifications, and adherence to prescribed therapies.
8. Medication initiation based on national formulary guidelines: Initiate lipid-lowering medications in accordance with the Thailand National List of Essential Medicines, taking into account health economics to align with the country's healthcare system. Regardless of the patient's medication reimbursement rights, physicians should prioritize the use of medications from this list, starting with generic options. If the target LDL-C level is not achieved, adjust the dosage accordingly. In the event of side effects or failure to reach treatment goals, consider switching or adding medications outside the Thailand National List of Essential Medicines, as appropriate. The use of multiple lipid-lowering agents should be guided by necessity, anticipated benefits, and safety, adhering to evidence-based practices.
9. Use of medications outside the Thailand National List of Essential Medicines: The use of medications outside the Thailand National List of Essential Medicines is subject to specific guidelines. It is important to review the indications, benefits, side effects, and risks. Currently, most lipid-lowering medications are available as generic drugs at lower prices, increasing patient access. If treatment needs to exceed the hospital's capabilities, consider referring patients to specialized care.
10. Monitoring treatment outcomes: Regularly monitor treatment outcomes, including potential drug side effects and the occurrence of ASCVD. Patients should be made aware that follow-up is necessary, as lipid-lowering drugs can cause side effects, and cardiovascular events can still occur despite medication, necessitating adjustments in treatment.
11. Dose adjustment or discontinuation of medication: There may be instances where it is necessary to reduce the dose or discontinue lipid-lowering medications temporarily or permanently. This may be considered in the following situations:
  - Adverse drug reactions, such as myopathy or rhabdomyolysis from statin use (Appendix 1).
  - Drug interactions with other medications cannot be avoided.
  - Impaired liver or kidney function (Appendix 2).
  - In primary prevention, lipid-lowering medications should not be initiated if the potential benefits are outweighed by the risks, such as in terminally ill patients.

## Recommendations for non-pharmacological treatment of dyslipidemia

Lifestyle modification as non-pharmacological management of dyslipidemia, which includes dietary management, exercise, and cigarette cessation, should be applied to all phases of treatment, not only as the initial treatment without medications but also as a combination with medications.

### General dietary recommendations

1. It is strongly recommended that the patients consume a diet consisting of vegetables, fruits, unrefined grains, and protein sources derived from fish, seafood, poultry, and low-fat dairy products. The intake of red meat, processed meat, sweets, and sugar-sweetened beverages should be reduced. This dietary approach aligns with the principles of Dietary Approaches to Stop Hypertension (DASH) and Mediterranean diets [11,12,13,14,15] [++, I, A].
2. Caloric restriction is strongly recommended for weight reduction in overweight or obese patients [16] [++, I, A].

### Dietary recommendations for LDL-C reduction

1. It is strongly recommended to reduce saturated fat intake, including sources such as animal fat, lard, palm oil, coconut oil, and butter, to <7% of total daily caloric intake [12, 13, 17, 18] [++, I, A].
2. It is strongly recommended to replace saturated fats with unsaturated fats rich in mono- or polyunsaturated fatty acids, such as olive oil, rice bran oil, and soybean oil [++, I, A].
3. It is strongly recommended to avoid the intake of trans fats, which are commonly found in margarine, shortening, and baked goods made with these ingredients [13, 16, 19] [++, I, A].
4. It is strongly recommended to increase the intake of dietary fiber [16, 20, 21] [++, I, A].
5. It is recommended to use dietary supplements containing 2 g/d of plant stanols [16, 22, 23] [+, IIa, A].

### Dietary recommendations for TG reduction

1. It is strongly recommended to abstain from consuming alcoholic beverages [16, 24] [++, I, A].
2. It is strongly recommended to reduce carbohydrate intake [16, 25,26,27] [++, I, A].
3. It is recommended to reduce sugar intake to <10% of total daily caloric intake [11, 28] [+, IIa, B].
4. In patients with high TG due to familial chylomicronemia syndrome, it is recommended to follow a very low-fat diet (fat intake <10–15 g/d) and to consume 30 g/d of medium-chain triglyceride oil [29, 30] [+, IIa, C].

### Exercise and smoking cessation

1. It is strongly recommended to engage in moderate-intensity aerobic exercise such as brisk walking, jogging, swimming, or cycling, for 150–300 min/week to help reduce LDL-C and TG, and increase HDL-C [31, 32] [++, IIa, A].
2. It is strongly recommended to quit smoking or refer patients to a smoking cessation clinic, as smoking is the most important preventable risk factor of ASCVD. Additionally, quitting smoking has the potential to increase HDL-C [33] [++, I, B].

## Recommendations for statin treatment of dyslipidemia

A recent systematic review on statin use for the primary prevention of cardiovascular disease published in 2022 [34] found that statin therapy was significantly associated with a reduced risk of total mortality (risk ratio [RR], 0.92 [95% confidence interval (CI), 0.87–0.98] with an absolute risk difference [ARD], −0.35% and a number needed to treat [NNT], 286), stroke (RR, 0.78 [95% CI, 0.68–0.90]; ARD, −0.39%; NNT, 256), myocardial infarction (RR, 0.67 [95% CI, 0.60–0.75]; ARD, −0.85%; NNT, 118), and composite cardiovascular outcomes (RR, 0.72 [95% CI, 0.64–0.81]; ARD, −1.28%; NNT, 78). The benefits of statin therapy were consistent across various groups defined by age, sex, race, ethnicity, lipid levels, cardiovascular risk score, and the presence of conditions such as hypertension, diabetes, metabolic syndrome, or kidney dysfunction. Notably, the benefits were most pronounced in individuals at high risk for cardiovascular events [34,35,36,37]. However, evidence regarding primary prevention in those over the age of 75 years is relatively sparse [38, 39]. In the context of secondary prevention, statin use is associated with a reduction in all-cause mortality by 18%–30%, with the greatest reductions observed in studies conducted in Asia [40, 41].

Statin use was not significantly associated with serious adverse events (RR, 0.97 [95% CI, 0.93–1.01]) [34]. Although 10%–15% of patients developed myalgia after statin use [42, 43], randomized controlled trials and systematic reviews did not find a significant association with the risk of myalgia (RR, 0.98 [95% CI, 0.86–1.11]) or elevation of alanine aminotransferase (ALT) level (RR, 0.94 [95% CI, 0.78–1.13]) [34]. In addition, statin use was not significantly associated with myopathy (RR, 1.09 [95% CI, 0.48–2.47]) or rhabdomyolysis (RR, 1.54 [95% CI, 0.36–6.64]) [34]. However, it is recommended that muscle symptoms should be monitored in patients on long-term use of statin.

### Primary prevention for individuals without diabetes or CKD (Figure 1)

1. For individuals aged 21 years or older with LDL-C >190 mg/dL, it is strongly recommended to target an LDL-C level of <100 mg/dL and to achieve at least a 50% reduction from baseline LDL-C prior to treatment [++, I, A]. Initiation of moderate-intensity statin therapy is strongly recommended; if the target is not achieved within 4–12 weeks, switching to high-intensity statin therapy is advised [7, 44] [++, I, A].
2. For individuals with FH, it is strongly recommended to target an LDL-C level of <70 mg/dL and to achieve at least a 50% reduction from baseline LDL-C prior to treatment [++, I, A]. Initiating high-intensity statin therapy is strongly recommended [++, I, A]. If the target is not achieved within 4–12 weeks, adding ezetimibe is recommended [+, I, A]. If the target remains unmet with ezetimibe, referral for consideration of proprotein convertase subtilisin/kexin type 9 (PCSK9) inhibitor therapy is recommended [2, 7, 8, 44] [+, IIa, A].
3. For individuals aged 35 years or older with LDL-C <190 mg/dL and a high cardiovascular risk, as assessed by the Thai CV Risk Score (10-year risk >10%), it is strongly recommended to target an LDL-C level of <100 mg/dL and to achieve at least a 30% reduction from baseline LDL-C prior to treatment [++, I, A]. Initiating low- to moderate-intensity statin therapy is strongly recommended, with dose adjustments to the maximum tolerated level to achieve the target [7, 44] [++, I, A].
4. For individuals aged 35 years or older with LDL-C <190 mg/dL and a Thai CV Risk Score of <10%, but who exhibit evidence of subclinical atherosclerosis (e.g., coronary calcium score >100 Agatston units or ankle-brachial index <0.9), a family history of premature cardiovascular disease, or chronic inflammation (e.g., psoriasis, rheumatoid arthritis, and HIV infection), low- to moderate-intensity statin therapy may be recommended. The target should be to reduce LDL-C to <100 mg/dL and achieve at least a 30% reduction from baseline, with dose adjustment to the maximum tolerated level to reach the target [7, 44] [+/−, IIb, B].
5. For individuals aged 35 years or older with elevated LDL-C levels <190 mg/dL, a Thai CV Risk Score of <10%, and no evidence of subclinical atherosclerosis, it is strongly recommended that patients consistently implement lifestyle modifications to effectively manage LDL-C levels [7, 44] [++, I, A].

**Figure 1.**
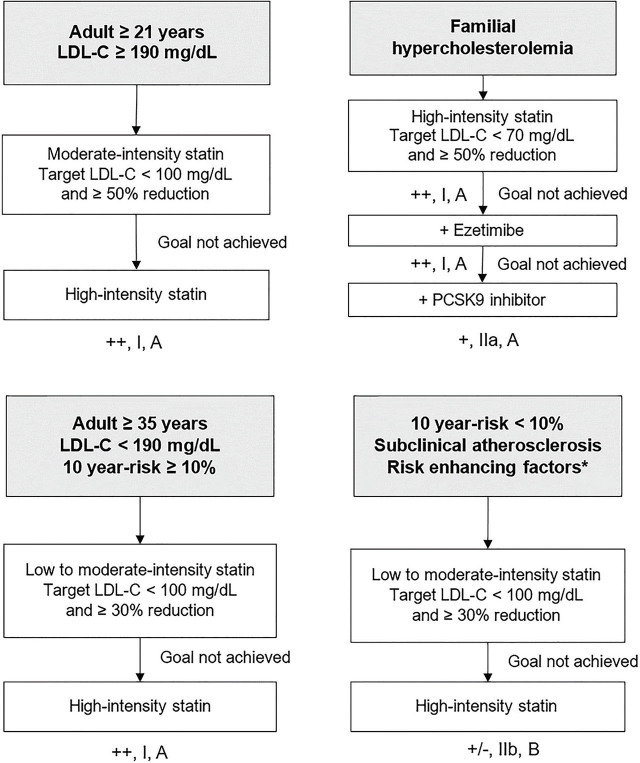
Primary prevention in individuals without diabetes or CKD. ^*^Risk-enhancing factors include evidence of subclinical atherosclerosis, a family history of premature cardiovascular disease, and chronic inflammation (e.g., psoriasis, rheumatoid arthritis, and HIV infection). CKD, chronic kidney disease; LDL-C, low-density lipoprotein-cholesterol; PCSK9, proprotein convertase subtilisin/kexin type 9.

### Primary prevention for individuals with diabetes mellitus (DM) (Figure 2)

1. For individuals with type 2 diabetes aged >40 years who have none or one risk factor for ASCVD, it is strongly recommended to initiate statin therapy in combination with lifestyle modifications to reduce LDL-C levels by at least 30% from baseline and to achieve a target LDL-C level of <100 mg/dL [++, I, A]. For patients with LDL-C levels >190 mg/dL, the goal should be to reduce levels by >50% from baseline. If increasing the statin dose is intolerable and the target of LDL-C level is not achieved, it is recommended to add ezetimibe [7, 45] [+, I, A].
2. For individuals with type 2 diabetes aged >40 years who have two or more risk factors for ASCVD such as duration of diabetes >10 years, being overweight or obese, smoking, hypertension, family history of premature ASCVD, CKD, or albuminuria [46], it is strongly recommended to initiate statin therapy in combination with lifestyle modification to achieve a reduction in LDL-C levels of at least 50% from baseline and to reach a target LDL-C level of <70 mg/dL [++, I, A]. If increasing the statin dose is intolerable and the target of LDL-C level is not achieved, adding ezetimibe is recommended [7, 45] [+, I, A]. If the target LDL-C level is still not achieved with statin and ezetimibe, PCSK9 inhibitors should be recommended [41] [+, I, A].
3. For individuals with type 2 diabetes aged <40 years who have none or one risk factor for ASCVD, lifestyle modifications should be recommended. If the LDL-C level remains >100 mg/dL after 3–6 months of lifestyle modification, statin therapy may be recommended, with a target LDL-C level of <100 mg/dL [7, 45] [+/−, IIb, C].
4. For individuals with type 2 diabetes aged <40 years who have two or more risk factors for ASCVD, lifestyle modification is strongly recommended. If the LDL-C level is >100 mg/dL after 3–6 months of lifestyle modification, statin therapy is recommended, with a target LDL-C level of <100 mg/dL [7, 45] [+, IIa, C].
5. Combination of statin and fibrate or niacin therapy in patients with high TG levels is not recommended, as it has not been shown to improve ASCVD outcomes compared with statin therapy alone [47, 48] [−, III, A].
6. For individuals with type 2 diabetes aged >40 years with two or more risk factors of ASCVD and have achieved the target LDL-C level with statin therapy, but have elevated TG levels, it is recommended to add pure eicosapentaenoic acid (EPA), such as icosapent ethyl, to reduce ASCVD risk [49] [+, IIa, B].

**Figure 2.**
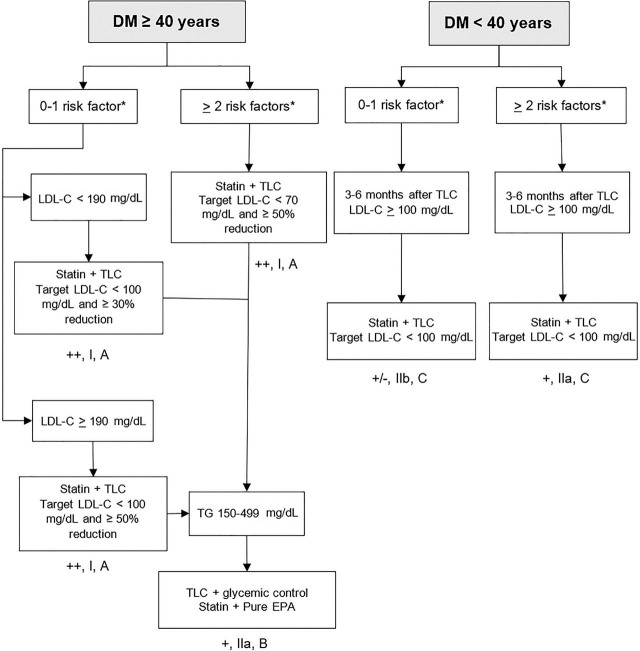
Primary prevention for individuals with DM. ^*^Risk factors include long-duration diabetes, obesity or overweight, smoking, hypertension, family history of premature cardiovascular diseases, CKD, and albuminuria. CKD, chronic kidney disease; DM, diabetes mellitus; EPA, eicosapentaenoic acid; LDL-C, low-density lipoprotein-cholesterol; TLC, therapeutic lifestyle changes.

### Primary prevention for individuals with CKD (Figure 3)

1. For individuals aged ≥50 years with CKD and an estimated glomerular filtration rate (eGFR) of <60 mL/min/1.73 m^2^ (CKD stages 3a–5), who are not yet on chronic dialysis or kidney transplantation (KT) and have an LDL-C level >100 mg/dL, it is strongly recommended to target an LDL-C level of <100 mg/dL or to achieve at least a 30% reduction from baseline levels [++, I, A]. The use of low- to moderate-intensity statins or a statin/ezetimibe combination is also strongly recommended [50,51,52] [++, I, A].
2. For individuals with albuminuria >30 mg/d or 30 mg/g of creatinine and an eGFR of ≥60 mL/min/1.73 m^2^ (CKD stages 1–2), who also have additional ASCVD risk factors, it is recommended to follow lipid-lowering management guidelines consistent with those for the general population [50,51,52] [+, I, A].
3. For individuals with CKD who have undergone KT, it is recommended to administer statins at an appropriate dosage, regardless of the target LDL-C level [50,51,52] [+, IIa, B].
4. Caution should be exercised when using high-intensity statins in patients with declining kidney function (CKD stages 3b–5). The choice of statin should strongly consider the safety data for statins in CKD patients [++, I, A] (Appendix 2).
5. For individuals receiving renal replacement therapy through hemodialysis or peritoneal dialysis who have not previously been on lipid-lowering medication, it is not recommended to initiate statins or statin/ezetimibe for lipid reduction [50,51,52] [−, III, B]. However, if there is an indication for secondary prevention, it is recommended to follow the guidelines for secondary prevention [+, II, C].
6. For individuals with CKD who are already on statin therapy and progress to require renal replacement therapy, it is recommended to continue statin use, with dosage adjustments made as necessary [50,51,52] [+, IIa, C] (Appendix 2).

**Figure 3.**
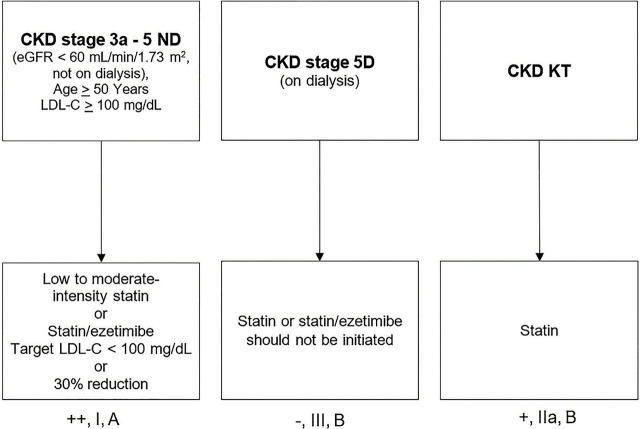
Primary prevention for individuals with CKD. CKD, chronic kidney disease; D, dialysis; ND, non-dialysis; KT, kidney transplantation; eGFR, estimated glomerular filtration rate; LDL-C, low-density lipoprotein-cholesterol.

### Secondary prevention for individuals with coronary syndromes (Figure 4)

1. For individuals with ACS, it is strongly recommended to initiate treatment with high-dose, high-intensity statin therapy as early as possible, unless contraindicated [2, 5, 7] [++, I, A]. It is strongly recommended to target LDL-C levels of <55 mg/dL and to achieve a reduction of at least 50% from the baseline LDL-C level prior to treatment [2, 5, 7] [++, I, A]. In cases where patients are intolerant to high-intensity statins or are over 75 years of age, moderate-intensity statins are strongly recommended [7] [++, I, A]. If the target LDL-C level is not achieved despite the highest tolerated statin dosage within 4–6 weeks, adding ezetimibe to the regimen is recommended [+, I, B]. If the target remains unmet despite the use of ezetimibe, consulting experts for consideration of PCSK9 inhibitors is recommended [2, 5, 7] [+, I, A].
2. For individuals with chronic coronary syndrome, statins are strongly recommended [++, I, A], with the dosage adjusted to the highest tolerable level to achieve the target LDL-C level. The goal is to achieve an LDL-C level of <70 mg/dL and a reduction of at least 50% from baseline LDL-C levels [++, I, A]. If feasible, aiming for an LDL-C level of <55 mg/dL is also recommended [2, 7, 53] [+, I, A]. If the target is not achieved within 4–12 weeks, adding ezetimibe is recommended [+, I, B]. If the target remains unmet despite the addition of ezetimibe, consulting experts for consideration of PCSK9 inhibitors is recommended [2, 7, 53] [+, I A].

**Figure 4.**
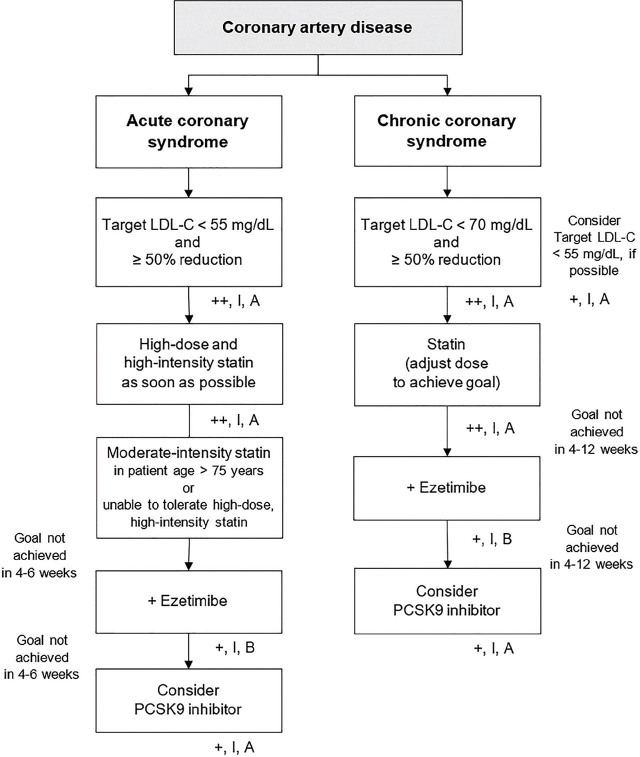
Secondary prevention for individuals with coronary syndromes. LDL-C, low-density lipoprotein-cholesterol; PCSK9, proprotein convertase subtilisin/kexin type 9.

### Secondary prevention for individuals with cerebrovascular diseases (Figure 5)

1. For individuals with ischemic stroke or TIA from a non-cardioembolic origin and LDL-C level ≥100 mg/dL, initiation of high-intensity statin therapy is strongly recommended [54,55,56] [++, I, B]. In cases where LDL-C levels are <100 mg/dL, moderate- to high-intensity statin therapy is recommended [+, IIa, C].
2. For individuals with ischemic stroke or TIA due to large vessel atherosclerotic disease, characterized by moderate stenosis (>50%) of the intracranial or extracranial carotid artery, it is strongly recommended to initiate high-intensity statin therapy in combination with ezetimibe, if needed, to achieve a target LDL-C level of <70 mg/dL [54, 56] [++, I, B]. If the target LDL-C level remains unmet despite the use of maximally tolerated statin combined with ezetimibe, and the patients are at very high risk of ASCVD events, consulting experts for consideration of PCSK9 inhibitors therapy is recommended [56,57,58,59] [+, IIa, B].
3. For individuals with ischemic stroke or TIA of cardioembolic origin, there is insufficient data from randomized controlled trials regarding the use of statins. However, descriptive studies suggest a potential benefit of initiating statin therapy. It may be reasonable to consider statin treatment if other cardiovascular risk factors are present [60] [+/−, IIb, C].
4. Statin therapy is not recommended for the prevention of recurrent hemorrhagic stroke in patients who have had a hemorrhagic stroke and do not have other indications for statin use [61, 62] [−, III, C].

**Figure 5.**
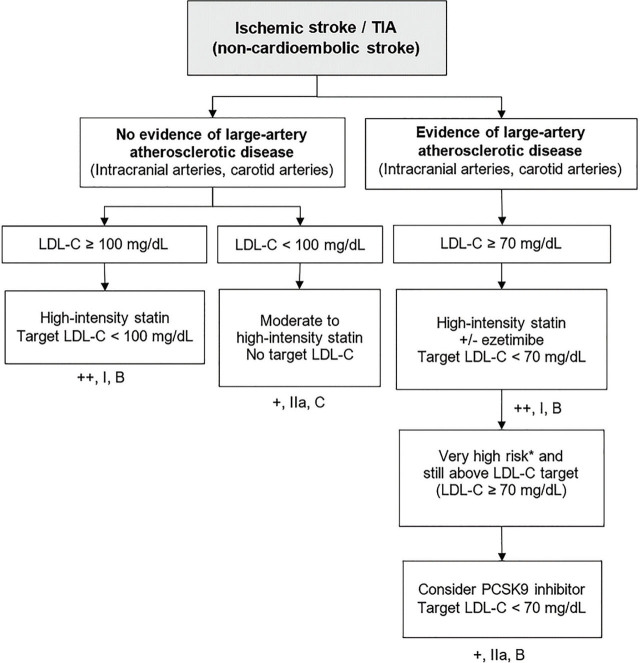
Secondary prevention for individuals with cerebrovascular diseases. ^*^Very high risk: stroke plus another major ASCVD (recent ACS, history of myocardial infarction, symptomatic peripheral arterial disease) or stroke plus multiple high-risk conditions (age ≥65 years, heterozygous FH, history of coronary artery bypass surgery or percutaneous coronary intervention outside of the major ASCVD events, diabetes, hypertension, CKD, current smoking). LDL-C, low-density lipoprotein-cholesterol; PCSK9, proprotein convertase subtilisin/kexin type 9; TIA, transient ischemic attack.

### Major ASCVD:

- Recent ACS (within the past 12 months)
- History of myocardial infarction (other than a recent ACS event listed above)
- Symptomatic peripheral arterial disease (history of claudication with ankle-brachial index <0.85 or previous revascularization or amputation)

### High-risk conditions:

- Age ≥65 years
- Heterozygous FH
- History of coronary artery bypass surgery or percutaneous coronary intervention outside of the major ASCVD events
- Diabetes
- Hypertension
- CKD (eGFR, 1559 mL/min/1.73 m^2^)
- Current smoking

### Assessment and monitoring before and after statin treatment

1. Prior to initiating lipid-lowering medication, patients should have their fasting plasma lipid levels measured at least once. A follow-up blood test is recommended within 4–12 weeks after starting the medication to evaluate its effectiveness and assess patient adherence. The efficacy of statins in reducing LDL-C levels depends on the specific type and dosage of the medication. Due to the variability in individual patient responses, periodic monitoring of treatment outcomes is necessary, typically every 3–12 months.
2. Liver enzyme levels, specifically aspartate aminotransferase (AST) and ALT, should be assessed prior to initiating statin therapy to exclude the presence of severe hepatitis, which would contraindicate the use of statins.
3. For patients with indications for liver enzyme monitoring, such as those at high risk for hepatitis or those with abnormal liver enzymes prior to starting statins, liver enzyme levels should be checked 4–12 weeks after initiating the medication or following any changes in dosage or type of statins to monitor for potential complications. Routine retesting is generally not necessary unless symptomatic liver disease is suspected or there are other specific indications for further testing.
4. If the reduction in LDL-C does not meet the target, it is essential to first evaluate medication adherence and recommend lifestyle modifications before making any adjustments to the treatment regimen.
5. If liver enzyme tests were not conducted prior to initiating statin therapy and enzyme levels (AST, ALT) are subsequently found to exceed 3 times the upper normal limit, it may be advisable to temporarily discontinue statins to investigate the underlying cause.
6. In cases of muscle pain, frequent cramps, or aches, testing for muscle enzymes is recommended. It is important to recognize that these symptoms, along with elevated creatine kinase (CK) levels, may not always be attributable to statin use. A temporary discontinuation of the statins, followed by reevaluation after 2–4 weeks, may be appropriate. If symptoms or abnormal test results recur upon reintroducing the same statin at the same or lower dose, it is likely that the symptoms are related to the statin.
7. If muscle pain or weakness is accompanied by CK levels >10 times the upper normal limit, this may suggest rhabdomyolysis, and statins should be discontinued immediately. A comprehensive review of the patient's statin use and other medications is essential. Statins may be reintroduced after symptoms resolve and CK levels normalize, with a reduced dosage as appropriate for the individual patient.
8. Statins should be considered the first-line treatment for patients requiring lipid-lowering therapy. If abnormal lipid levels persist despite tolerating the maximum dose of statins, additional non-statin lipid-lowering medications should be considered. Treatment outcomes and side effects should be closely monitored (Appendix 1).

## Recommendations for using non-statin lipid-lowering medications

A number of non-statin lipid-powering medications are available for clinical use. These include fibrates, ezetimibe, PCSK9 inhibitors, bempedoic acid, cholestyramine, and fish oil (omega-3 fatty acids). The main clinical cardiovascular outcome studies of these non-statin medications have been summarized followed by recommendations for their use.

The Fenofibrate Intervention and Event Lowering in Diabetes (FIELD) study, which compared fenofibrate with placebo in diabetic patients, found no significant benefit in reducing cardiovascular disease with fenofibrate [63]. Similarly, the Action to Control Cardiovascular Risk in Diabetes Study Group (ACCORD)-LIPID study, which evaluated the combination of statins and fenofibrate versus statin alone in patients with type 2 diabetes, did not demonstrate a significant reduction in cardiovascular disease with the addition of fenofibrate [47]. However, both FIELD and ACCORD-LIPID studies observed that fenofibrate reduced the risk of diabetic retinopathy [64, 65].

Studies have demonstrated the cardiovascular benefits of ezetimibe. The Improved Reduction of Outcomes: Vytorin Efficacy International Trial (IMPROVE-IT) investigated the effects of combining ezetimibe (10 mg/d) with statins in patients with ACS, revealing a 7% reduction in cardiovascular events compared to those treated with statins alone [57]. Additionally, the Study of Heart and Renal Protection (SHARP) found that combining simvastatin (20 mg/d) with ezetimibe in patients with CKD resulted in a 17% reduction in major atherosclerotic events compared to the placebo group over approximately 5 years of follow-up [51].

The FOURIER (Further Cardiovascular Outcomes Research with PCSK9 Inhibition in Subjects with Elevated Risk) study, a cardiovascular outcome trial for evolocumab, enrolled 27,564 patients with ASCVD and plasma LDL-C levels ≥70 mg/dL who had previously been treated with statins. Evolocumab, administered as 140 mg subcutaneously (SC) every 2 weeks or 420 mg SC every 4 weeks, achieved a 59% reduction in plasma LDL-C levels and a 15% reduction in major adverse cardiovascular events (MACE) compared to placebo over an average follow-up of 2.2 years [58]. The median LDL-C level decreased from 92 mg/dL to 30 mg/dL. Side effects included injection site rash in 2.1% of patients versus 1.6% in the placebo group. There were no significant differences in the rates of diabetes or neurocognitive impairment between the treatment and placebo groups.

The ODYSSEY OUTCOMES (Evaluation of Cardiovascular Outcomes After an Acute Coronary Syndrome During Treatment With Alirocumab) study, which involved 18,924 patients with ACS, compared alirocumab (75 mg or 150 mg SC every 2 weeks) with placebo. Over a period of 2.8 years, alirocumab reduced the incidence of MACE by 15% compared to placebo [59]. Additionally, a patient-level analysis of ORION-9, 10, 11, which included 3,655 patients followed for approximately 18 months, demonstrated that inclisiran, a PCSK9 siRNA, reduced the rate of MACE by 25% compared to the placebo [66]. The cardiovascular outcomes trial of inclisiran, named ORION-4, is currently underway.

The Cholesterol Lowering via Bempedoic Acid (CLEAR) Outcome study evaluated the cardiovascular effects of bempedoic acid in 13,970 statin-intolerant patients at high risk for cardiovascular disease or with a history of cardiovascular events. With a baseline average LDL-C level of 139 mg/dL, the study found that treatment with bempedoic acid (180 mg/d) significantly reduced the incidence of MACE by 13% compared to placebo over an average follow-up of 40.6 months [67].

The Reduction of Cardiovascular Events with Icosapent Ethyl-Intervention Trial (REDUCE-IT), which included 8,179 individuals at high risk for ASCVD, assessed the impact of 4 g/d icosapent ethyl, an ethyl ester of EPA versus mineral oil over 4.9 years. Icosapent ethyl was associated with a 25% reduction in ASCVD risk [49]. In contrast, the STRENGTH (Long-Term Outcomes Study to Assess Statin Residual Risk with Epanova in High Cardiovascular Risk Patients with Hypertriglyceridaemia) study, involving 13,078 individuals at moderate to high ASCVD risk, evaluated the effects of 4 g/d of EPA (75%) and docosahexaenoic acid (DHA; 25%) combination compared to corn oil over 3.5 years. This study found no significant benefit in ASCVD risk reduction with the EPA + DHA combination [68].

Prior to the widespread use of statins, bile acid sequestrants were shown to reduce the incidence of cardiovascular disease. In a study involving 3,806 men with hypercholesterolemia, cholestyramine administered at a dose of 24 g/d demonstrated a 19% reduction in non-fatal myocardial infarction over 7.4 years compared to placebo. This benefit was associated with a reduction in plasma LDL-C levels [69].

### Fibrates

1. It is strongly recommended to initiate fibrate therapy in conjunction with lifestyle modifications for the prevention of acute pancreatitis in patients with fasting plasma TG levels >500 mg/dL [70, 71] [++, I, A].
2. Combining fibrates with statins does not provide additional cardiovascular risk reduction benefits beyond those provided by statin therapy alone. However, fenofibrate may be beneficial in slowing or reducing the progression of diabetic retinopathy and decreasing the need for laser interventions in patients with this condition [64, 65] [+, IIa, B].
3. The use of gemfibrozil in combination with statins is not recommended due to the increased risk of adverse effects [−, III, B].
4. When prescribing fibrates, it is crucial to assess and consider renal function (Appendix 2).

### Ezetimibe

1. Ezetimibe monotherapy is not recommended for the reduction of LDL-C [−, III, C]. It should be used in combination with statins unless statin therapy is intolerable.
2. Ezetimibe in combination with statins is recommended in the following cases:
  - For patients who, despite being on the maximum tolerated dose of statin for at least 2–3 months, have LDL-C levels that remain above target. This is applicable to high-risk patients for both primary and secondary prevention [2, 57] [+, I, A].
  - For patients whose LDL-C levels are above target but who are unable to tolerate high-dose statin therapy, such as those with stage 4–5 CKD who are not on dialysis [+, I, A].
3. Switching from statin therapy to ezetimibe alone is not recommended if liver enzyme levels (AST, ALT) are elevated but not exceeding 3 times the upper normal limit, and no other causes have been identified [−, III, C].

### PCSK9 inhibitors (PCSK9 mAb and PCSK9 siRNA)

1. PCSK9 inhibitors in combination with statins and ezetimibe are recommended for patients with established ASCVD and plasma LDL-C levels ≥70 mg/dL [7, 58, 59, 66] [+, I, A].
2. PCSK9 inhibitors in combination with ezetimibe are recommended for patients with ASCVD or those at high risk for cardiovascular disease with plasma LDL-C levels above target in patients with statin intolerance [7] [+, I, A].
3. PCSK9 inhibitors in combination with statins and ezetimibe are recommended for patients with FH and plasma LDL-C levels ≥100 mg/dL [7] [+, IIa, A].
4. PCSK9 inhibitors in combination with statins and ezetimibe are recommended for diabetic patients at high risk for cardiovascular disease who are on the maximum tolerated dose of statins and ezetimibe, with plasma LDL-C levels remaining above target [72] [+, I, A].

### Bempedoic Acid

1. Bempedoic acid in combination with ezetimibe is recommended for patients who experience adverse effects from statins and have plasma LDL-C levels that remain above target despite treatment with ezetimibe alone [67] [+, IIb, B].

### Cholestyramine

1. Cholestyramine is recommended in combination with statins and ezetimibe for patients with ASCVD or those at high risk for cardiovascular disease who have LDL-C levels above target and are unable to use PCSK9 inhibitors [+, IIb, C].
2. It is strongly recommended to administer cholestyramine at least 4 h apart from other medications, or alternatively, to take other medications at least 1 h before cholestyramine [++, I, C].
3. Cholestyramine is strongly not recommended for patients with plasma TG levels >500 mg/dL [71] [––, III, C].

### Fish oil (Omega-3 fatty acids)

1. Omega-3 fatty acids (EPA/DHA, pure EPA) in doses >2 g/d are recommended to lower plasma TG levels when plasma TG levels >500 mg/dL in patients who cannot take fibrates, or to be used in combination with fibrates in patients with severe hypertriglyceridemia [70] [+, IIa, A].
2. Pure EPA at 4 g/d is recommended for patients at high risk for cardiovascular disease who still have high plasma TG levels (150–499 mg/dL) after prescribing statin therapy to reduce cardiovascular events [73] [+, IIa, B].

## Appendix 1

The types and administration of medications for treating dyslipidemia.

**Table A1.1.** Classification of statins by LDL-C reduction efficacy

**High-intensity statin**	**Moderate-intensity statin**	**Low-intensity statin**
Reduces LDL-C by >50% before treatment	Reduces LDL-C by approximately 30%–50% before treatment	Reduces LDL-C by <30% before treatment

Atorvastatin 40–80 mg/d Rosuvastatin 20 mg/d	Atorvastatin 10–20 mg/d Fluvastatin 80 mg/d Pitavastatin 1–4 mg/d Pravastatin 40 mg/d Rosuvastatin 5–10 mg/d Simvastatin 20–40 mg/d	Fluvastatin 20–40 mg/d Pravastatin 10–20 mg/d Simvastatin 10 mg/d

CKD, chronic kidney disease; LDL-C, low-density lipoprotein-cholesterol.

Notes:
Individual responses to statin medications may vary. Some patients may respond well even at lower than recommended doses. Simvastatin 80 mg/d should not be used. Simvastatin should not be used in combination with azole antifungals, erythromycin, clarithromycin, HIV protease inhibitors, gemfibrozil, cyclosporine, or danazol. Simvastatin should not >10 mg/d when used with verapamil or diltiazem. Simvastatin should not >20 mg/d when used with amiodarone, amlodipine, or ranolazine. Caution is advised when using high-intensity statins in patients over 75 years old, those at risk of drug interactions, patients with a history of cerebral hemorrhage (not due to trauma), and those with CKD stages 3b–5.

**Table A1.2.** Medications for dyslipidemia treatment

**Medications**	**Recommended dosage**	**Efficacy**	**Side effects**
**LDL-C lowering medications**			
Statins	Atorvastatin 10–80 mg/d Fluvastatin 20–80 mg/d Lovastatin 10–80 mg/d Pitavastatin 1–4 mg/d Pravastatin 10–80 mg/d Rosuvastatin 5–20 mg/d Simvastatin 10–40 mg/d (Take once daily, preferably after dinner for simvastatin)	Lower plasma LDL-C levels by 21%–55% Increases plasma HDL-C by 2%–10% Lower plasma TG levels by 6%–30%	- Risk of muscle symptoms, hepatitis, and increased diabetes risk. - Simvastatin and atorvastatin may interact with CYP450 inhibitors. - Maximum dose of simvastatin is 40 mg/d; avoid using 80 mg/d.
Ezetimibe (cholesterol absorption inhibitor)	Ezetimibe 10 mg/d (Take once daily)	Lowers plasma LDL-C by 10%–18% and apoB by 11%–16%. Combined with statins, reduces LDL-C by an additional 25% [69].	- May cause muscle symptoms.
Bile acid sequestrants	Cholestyramine 8–16 mg/d	Lowers plasma LDL-C levels by 15%–25%	- Not recommended for use in mixed dyslipidemia as it can increase plasma TG levels. - Can cause gastrointestinal symptoms. - Reduces the absorption of fat-soluble vitamins. - Interferes with the absorption of other medications, including statins, so other medications should be taken 1 h before or 4 h after cholestyramine.
PCSK9 monoclonal antibody	Alirocumab 75 mg or 150 mg SC every 2 weeks, or 300 mg every 4 weeks Evolocumab 140 mg SC every 2 weeks, or 420 mg every 4 weeks.	Lowers plasma LDL-C levels by 50%–60%	- Injection site reaction, flu-like symptoms. - Keep in a cooler device with a cold pack to maintain drug efficacy during transportation in tropical climates [71].
PCSK9 siRNA	Inclisiran 248 mg SC at 0, 3, then every 6 months	Lowers plasma LDL-C levels by 40%–60%	- Injection site reaction, headache, bronchitis.
Bempedoic acid	180 mg/d	Lowers plasma LDL-C levels by 23% when used as monotherapy, 36% when combined with ezetimibe, and 12.6%–16.5% when combined with statins	- Gout, flu-like symptoms, tendon injury.
**Triglyceride-lowering medications**			
Fibrates	Fenofibrate 100, 300 mg/d Micronized fenofibrate 200 mg/d Microcoated fenofibrate 160 mg/d Nanoparticle fenofibrate 145 mg/d Fenofibric acid (delayed release) 135 mg/d Fenofibric acid 145, 160, 200 mg/d Gemfibrozil 600–1,200 mg/d Pemafibrate 0.2–0.4 mg/d	Lower plasma TG levels by 20%–35% Increases plasma HDL-C by 6%–18%	- Gemfibrozil: not recommended with statins. - Fenofibrate: take with food to enhance absorption. - Use fibrates with caution in CKD patients.
Omega-3 fatty acids	EPA/DHA 2–4 g/d Pure EPA 2–4 g/d	Lower plasma TG by 20%–50%	- Increased bleeding risk.

CKD, chronic kidney disease; DHA, docosahexaenoic acid; EPA, eicosapentaenoic acid; HDL-C, high-density lipoprotein-cholesterol; LDL-C, low-density lipoprotein-cholesterol; PCSK9, proprotein convertase subtilisin/kexin type 9; SC, subcutaneously; TG, triglycerides.

## Appendix 2

### Definition of chronic kidney disease (CKD)

A CKD patient is defined as an individual who meets at least one of the following 2 criteria:

1. A patient with abnormal kidney function lasting for >3 months, regardless of whether the estimated glomerular filtration rate (eGFR) is abnormal. Abnormal kidney function is defined by one or more of the following criteria:
  1.1 At least 2 occurrences of the following abnormalities within a 3-month period:
    - Presence of albumin in the urine (albuminuria), with an albumin excretion rate (AER) of >30 mg/24 h or an albumin-to-creatinine ratio (ACR) of >30 mg/g.
    - Presence of red blood cells in the urine (hematuria).
    - Abnormal electrolyte levels caused by dysfunction of the renal tubules.
  1.2 Detection of abnormalities through radiological imaging.
  1.3 Detection of structural or pathological abnormalities.
  1.4 A history of kidney transplant surgery.
2. A patient with an eGFR of <60 mL/min/1.73 m^2^ for >3 months, regardless of whether abnormal kidney function is detected or not.

### Classification of CKD

1. CKD stages should be classified based on the cause, eGFR level (Table A2.1), and the amount of albumin in the urine (Table A2.2).
2. The cause and type of CKD should be classified according to systemic diseases, genetic diseases, environmental factors, and the anatomical structure or pathology of the kidneys.

**Table A2.1.** Stages of CKD

**CKD stage**	**eGFR (mL/min/1.73 m^2^)**	**Definition**
Stage 1	>90	Normal or high
Stage 2	60–89	Mild decrease
Stage 3a	45–59	Mild to moderate decrease
Stage 3b	30–44	Moderate to severe decrease
Stage 4	15–29	Severe decrease
Stage 5	<15	End-stage kidney failure

CKD, chronic kidney disease; eGFR, estimated glomerular filtration rate.

Notes:
If there is no evidence of kidney abnormalities, stages 1 and 2 do not meet the criteria for CKD diagnosis. When reporting eGFR calculations, round the number to the nearest whole number before classifying the CKD stage. For example, if a person's eGFR is 59.64 mL/min/1.73 m^2^, it should be rounded to 60 mL/min/1.73 m^2^. If this person has other kidney abnormalities, they would be classified as stage 2 CKD, but if there are no other abnormalities, they would not be considered to have CKD.

**Table A2.2.** Diagnostic criteria for albumin in urine

**Stage**	**AER (mg/24 h)**	**ACR (mg/mmol)**	**ACR (mg/g)**	**Definition**
A1	<30	<3	<30	Normal or slightly increased
A2	30–300	3–30	30–300	Moderately increased
A3	>300	>30	>300	Severely increased

ACR, albumin-to-creatinine ratio; AER, albumin excretion rate.

Notes:
Stage A3 includes patients with nephrotic syndrome (AER >2,200 mg/24 h or ACR >2,200 mg/g or >220 mg/mmol). If albumin in urine cannot be measured, a urine albumin strip test can be used as a substitute.

**Table A2.3.** Maximum recommended doses of lipid-lowering drugs in CKD patients

**Drug group and name**	**Maximum recommended dose by CKD stage**			
	**Stage 1–2 (GFR ≥60 mL/min/1.73 m^2^))**	**Stage 3a (GFR 45–59 mL/min/1.73 m^2^)**	**Stage 3b–5 (GFR <45 mL/min/1.73 m^2^)**	**Kidney transplant**
**Statin (mg/day)**				
Atorvastatin	40–80	40–80	20–40	20
Fluvastatin	80	80	No data	80
Pitavastatin	4	4	2	No data
Pravastatin	40	40	20	20
Rosuvastatin	40	20	10	5
Simvastatin	40	40	20–40	20
Simvastatin/ezetimibe	40/10	40/10	20/10	20/10

**Bile acid sequestrants (g/day)**				
Cholestyramine	16	16	16	16

**Fibric acid derivatives (mg/day)**				
Fenofibrate	300	100	Not recommended	Not recommended
Fenofibrate (micronized form)	200	100	Not recommended	Not recommended
Fenofibrate (micronized and microcoated form)	160	80	Not recommended	Not recommended
Fenofibrate (nano-technology form)	145	72.5	Not recommended	Not recommended
Gemfibrozil	1,200	1,200	600	600
Pemafibrate	0.4	0.4	0.2	No data

**Others (mg/day)**				
Ezetimibe	10	10	10	10
Niacin	2,000	2,000	1,000	No data
Omega-3 fatty acids (EPA/DHA)	4,000	4,000	4,000	4,000
Pure EPA	4,000	4,000	4,000	4,000
Alirocumab	75–150	75–150	75–150	75–150
Evolocumab	140–420	140–420	140–420	140–420
Inclisiran	284	284	284	284

CKD, chronic kidney; DHA, docosahexaenoic acid; EPA, eicosapentaenoic acid.
